# Hybutimibe, an innovative cholesterol absorption inhibitor for lipid-lowering therapy: current evidence and future perspectives

**DOI:** 10.3389/fphar.2026.1936459

**Published:** 2026-10-05

**Authors:** Sha Li, Hui-Hui Liu, Jian-Jun Li

**Affiliations:** Cardiometabolic Center, State Key Laboratory of Cardiovascular Disease, Fuwai Hospital, National Center for Cardiovascular Diseases, Chinese Academy of Medical Sciences, Peking Union Medical College, Beijing, China

**Keywords:** China, cholesterol absorption inhibitor, hybutimibe, LDL-c lowering, statin combination therapy

## Abstract

Achievement of low-density lipoprotein cholesterol (LDL-C) targets is crucial for the prevention of atherosclerotic cardiovascular disease (ASCVD). As first-line lipid-lowering drugs, statins often fail to achieve adequate LDL-C control rates in clinical practice due to individual variations in efficacy, safety concerns, and poor long-term adherence. Cholesterol absorption inhibition mediated by the Niemann-Pick C1-like 1 (NPC1L1) pathway provides a complementary approach to statin-based lipid lowering regimens. Hybutimibe is an oral NPC1L1 inhibitor approved in China and has reported pharmacokinetic characteristics that differ from those of ezetimibe; however, the clinical relevance of these differences has not been established in direct comparative studies. Evidence from Phase I-III clinical trials and post-marketing real-world studies indicates that hybutimibe monotherapy or combination therapy with statins can achieve significant and sustained LDL-C reduction; in patients with inadequate response to statin monotherapy, combination with hybutimibe exhibits superior lipid-lowering efficacy compared to statin dose escalation, with favorable overall safety. However, available studies have primarily evaluated LDL-C and other lipid parameters as surrogate endpoints. No dedicated cardiovascular outcome trial has established whether hybutimibe reduces major adverse cardiovascular events, cardiovascular mortality, or all-cause mortality. This article systematically reviews the research and development background, pharmacological properties, mechanism of action, clinical efficacy, safety, and post-marketing real-world evidence of hybutimibe, exploring its application value as an adjuvant therapy in optimizing clinical LDL-C management.

## 1 Introduction

Atherosclerotic cardiovascular disease (ASCVD) remains the leading cause of global disease burden and mortality. According to the Global Burden of Disease 2023 study, CVDs accounted for 19.2 million deaths and 437 million disability-adjusted life years (DALYs) worldwide in 2023, with the number of prevalent cases increasing to 626 million globally. Elevated low-density lipoprotein cholesterol (LDL-C) is firmly established as a causal and modifiable driver of ASCVD onset and progression (Global, 2025). Extensive epidemiologic, genetic, and randomized trial evidence demonstrates that sustained LDL-C lowering proportionally reduces ASCVD risk (Borén et al., 2020), forming the foundation of contemporary prevention strategies. International expert consensus emphasizes LDL-C as the primary therapeutic target and supports intensive lipid reduction to achieve greater clinical benefit (Borén et al., 2020). These principles are reflected in modern guidelines, which advocate increasingly stringent LDL-C goals for high-risk and very-high-risk populations (Mach, 2019).

Statins remain the cornerstone of LDL-C-lowering therapy and have consistently demonstrated substantial reductions in cardiovascular morbidity and mortality across diverse populations (Borén et al., 2020). However, real-world evidence indicates that a considerable proportion of patients fail to achieve guideline-recommended LDL-C targets with statin monotherapy (Gitt et al., 2016). In addition to interindividual variability in LDL-C response, dose escalation of statins is frequently constrained by safety and tolerability concerns, including elevations in liver enzymes and statin-associated muscle symptoms, which adversely affect treatment persistence and long-term adherence (Cheeley et al., 2022).These limitations have prompted increasing use of non-statin lipid-lowering therapies, such as cholesterol absorption inhibitors and proprotein convertase subtilisin/kexin type 9 (PCSK9)-targeted agents, as add-on or combination strategies to improve LDL-C target attainment in routine clinical practice (Mach, 2019).

Cholesterol absorption inhibitors act by blocking Niemann-Pick C1-like 1 (NPC1L1)-mediated intestinal cholesterol uptake. As the first clinically approved cholesterol absorption inhibitor, ezetimibe has demonstrated additional LDL-C lowering and cardiovascular benefit when added to statin therapy (Patrick et al., 2002). In the IMPROVE-IT trial involving patients with recent acute coronary syndrome, combination therapy with ezetimibe and simvastatin achieved a mean on-treatment LDL-C level of approximately 54 mg/dL, compared with about 70 mg/dL with simvastatin alone, and was associated with a significant reduction in cardiovascular events (Cannon et al., 2015). Evidence from the EWTOPIA 75 trial further supports absorption inhibition as an effective primary prevention strategy in elderly individuals without prior coronary artery disease (Ouchi et al., 2019).

Hybutimibe, a newer selective cholesterol absorption inhibitor developed in China, has shown promising lipid-lowering effects across Phase I-III clinical trials and post-marketing real-world studies (Wang and Xu, 2025; Yunxiang et al., 2025; Mu and Qiu, 2025; Wu et al., 2025; Ren and Chen, 2025; Xie et al., 2024; Qi et al., 2022a). In a multicenter, randomized Phase III study, add-on hybutimibe 20 mg achieved an additional 16.39% LDL-C reduction, exceeding the 6.75% reduction with atorvastatin dose doubling, with good tolerability (Qi et al., 2022a). Consistent with these findings, real-world evidence from high-risk populations, including coronary heart disease, atherosclerotic ischemic stroke, and chronic heart failure with hyperlipidemia, showed that hybutimibe-containing regimens improved LDL-C and related lipid parameters in routine clinical practice (Wang and Xu, 2025; Yunxiang et al., 2025; Mu and Qiu, 2025; Wu et al., 2025; Ren and Chen, 2025; Xie et al., 2024).

Hybutimibe has progressed from early pharmacological development to Phase I–III clinical evaluation, regulatory approval in China, and initial post-marketing use. However, the available evidence has not been comprehensively assessed in an international context that distinguishes pharmacokinetic characteristics from demonstrated clinical benefits and lipid surrogate endpoints from cardiovascular outcomes. A critical synthesis is therefore timely to define the current level of evidence and the remaining knowledge gaps.

Here, we review the contemporary landscape of LDL-C-lowering therapy and the unmet needs in achieving guideline-recommended targets; summarize the development, pharmacological characteristics, mechanism of action, clinical efficacy, safety, and post-marketing evidence of hybutimibe; and critically evaluate its potential clinical position in relation to established and emerging non-statin therapies.

## 2 Contemporary LDL-C-lowering therapies and the clinical context in China

Contemporary non-statin LDL-C-lowering therapies include NPC1L1 inhibitors, monoclonal antibodies targeting PCSK9, inclisiran, and bempedoic acid. These agents differ in mechanism, route and frequency of administration, magnitude of LDL-C lowering, cardiovascular outcome evidence, safety considerations, cost, and accessibility (Figure 1). Ezetimibe and PCSK9 monoclonal antibodies have demonstrated cardiovascular benefit in randomized outcome trials, while bempedoic acid has reduced major adverse cardiovascular events in statin-intolerant patients at high cardiovascular risk (Nissen et al., 2023). Inclisiran provides sustained LDL-C reduction with infrequent dosing, although definitive cardiovascular outcome data remain pending (Ray et al., 2020). Hybutimibe is an oral NPC1L1 inhibitor approved in China, but its current evidence base is derived predominantly from lipid efficacy trials and observational studies conducted in Chinese populations. Its clinical position should therefore be considered in relation to both established and emerging non-statin therapies.

**FIGURE 1:**
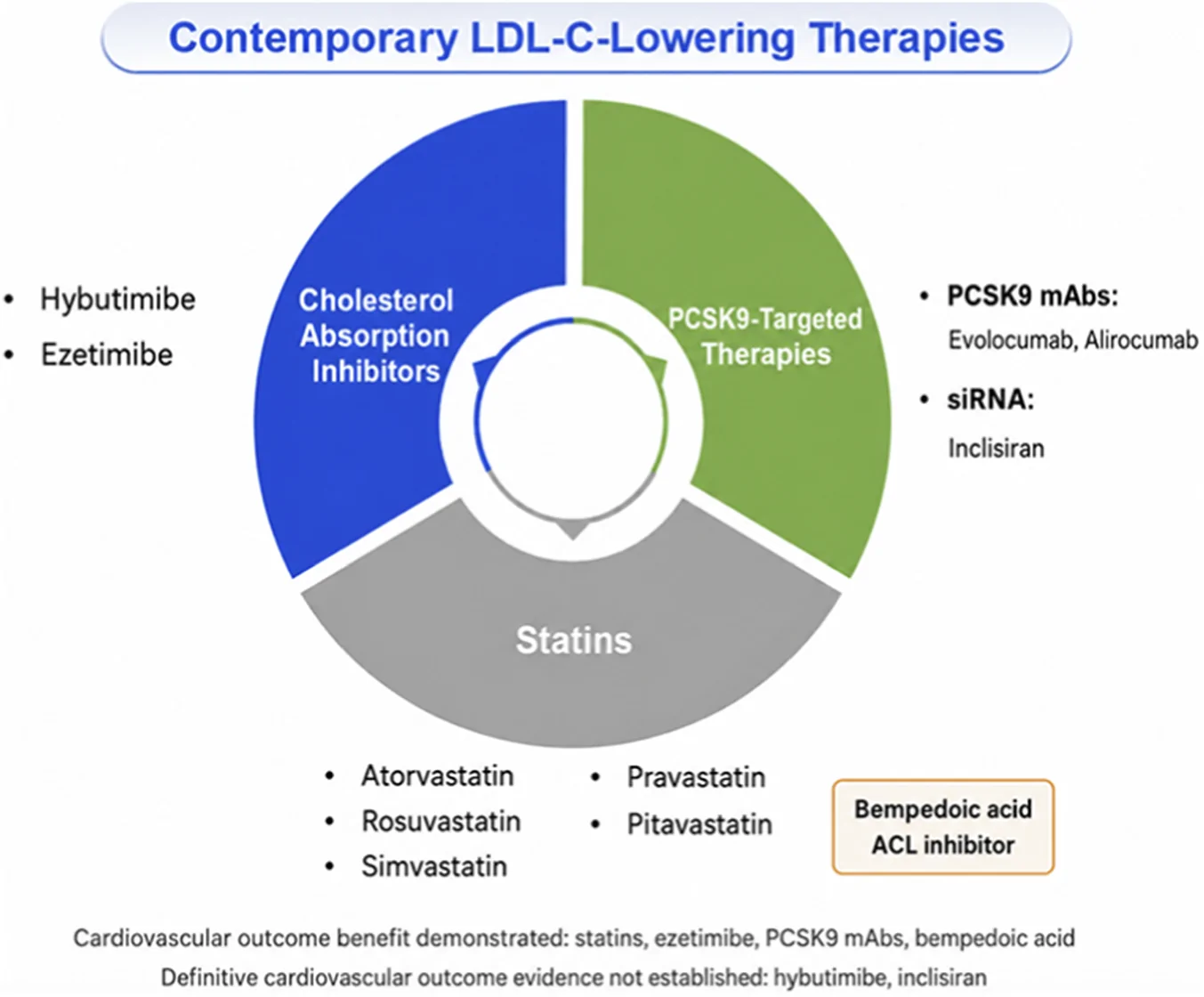
Mechanisms and Evidence Status of Contemporary LDL-C-Lowering Therapies. Statins inhibit hepatic cholesterol synthesis through HMG-CoA reductase, whereas bempedoic acid inhibits ATP-citrate lyase upstream of HMG-CoA reductase. Ezetimibe and hybutimibe reduce intestinal cholesterol absorption by targeting NPC1L1. PCSK9 monoclonal antibodies and inclisiran enhance LDL receptor availability through extracellular PCSK9 inhibition and hepatic PCSK9 synthesis inhibition, respectively. Cardiovascular outcome benefits have been demonstrated for statins, ezetimibe added to statin therapy, PCSK9 monoclonal antibodies, and bempedoic acid in selected populations. Hybutimibe currently has LDL-C-lowering efficacy data but no dedicated cardiovascular outcome trial, while cardiovascular outcome trials of inclisiran remain ongoing.

### 2.1 Statins: cornerstone therapy with persistent unmet needs

Statins remain the first-line and most widely used agents for LDL-C reduction in China. The Chinese Guideline for Lipid Management (2024) recommends statins as the preferred initial therapy for both primary and secondary prevention of ASCVD, consistent with international guidelines such as the 2018 ACC/AHA cholesterol guideline (Wilson et al., 2019; Joint Committee on the Chinese Guidelines for Lipid Management, 2024). Extensive randomized trials and meta-analyses have established statins as the foundation of lipid-lowering therapy, demonstrating robust reductions in LDL-C and cardiovascular risk across a broad range of populations.

According to the 2019 ESC/EAS Guidelines, LDL-C remains the primary therapeutic target, with recommended goals of <1.8 mmol/L for patients at high cardiovascular risk and <1.4 mmol/L together with a ≥50% reduction from baseline for those at very high risk (Mach, 2019). However, evidence from real-world studies indicates that these guideline-recommended LDL-C targets are often not achieved with statin monotherapy in routine clinical practice. Large registry studies, including the DAVINCI and DYSIS cohorts, have shown that a substantial proportion of high-risk patients fail to reach LDL-C goals even when treated with guideline-recommended statin regimens (Gitt et al., 2016). Statin dose escalation is often constrained by tolerability concerns, with statin intolerance affecting approximately 9% of treated individuals (Bytyci et al., 2022). Muscle-related symptoms are frequently reported in routine practice, while the incidence of significant elevation of alanine aminotransferase/aspartate aminotransferase (ALT/AST) (≥3 times the upper limit of normal) is <1% in patients on low-to-moderate doses, rising to ∼2%–3% in high-dose regimens (Stroes et al., 2015; Calderon et al., 2010). Severe muscle injury, such as rhabdomyolysis, remains rare, occurring in 3–4 cases per 100,000 patient-years (Catapano, 2012). Both true and perceived statin intolerance represent practical barriers to long-term adherence and effective LDL-C lowering. High-quality evidence indicates that partial or complete statin intolerance markedly limits achievable LDL-C reduction and often necessitates the use of non-statin lipid-lowering therapies (Cheeley et al., 2022).

### 2.2 Cholesterol absorption inhibitors: a rationale for combination therapy

Given the limitations of statin monotherapy and the restricted use of PCSK9 inhibitors, cholesterol absorption inhibitors have emerged as a practical and complementary option within the lipid-lowering treatment paradigm. By blocking NPC1L1-mediated intestinal cholesterol uptake, this class provides LDL-C reduction through a pathway distinct from hepatic cholesterol synthesis.

Multiple domestic and international guidelines, including the Chinese Guidelines on Dyslipidemia, the 2024 Chinese Expert Consensus on Lipid Management in Diabetes, and guidance on the management of mixed dyslipidemia, recommend early initiation of combination therapy when LDL-C targets cannot be achieved with statins alone (Wilson et al., 2019; Adults, 2016; Beijing Hypertension Association, 2022). The addition of a cholesterol absorption inhibitor is therefore considered an effective and guideline-supported strategy to further reduce LDL-C without substantially increasing safety concerns. The IMPROVE-IT trial demonstrated that adding ezetimibe to statin therapy resulted in additional LDL-C lowering and a reduction in cardiovascular events in patients with recent acute coronary syndrome (Cannon et al., 2015).

Hybutimibe is an oral NPC1L1 inhibitor approved in China and provides an additional option within the cholesterol absorption inhibitor class. A 2022 national expert consensus on the comanagement of coronary heart disease and ischemic stroke recommends consideration of cholesterol absorption inhibitors in patients who do not achieve LDL-C targets with statin therapy alone (Beijing Hypertension Association, 2022).

### 2.3 PCSK9 inhibitors: potent LDL-C reduction with practical limitations

PCSK9 inhibitors provide profound LDL-C-lowering efficacy and represent an important therapeutic option for patients at very high cardiovascular risk (Mach, 2019; Sabatine et al., 2017; Schwartz et al., 2018). By enhancing hepatic LDL receptor recycling and preventing LDL receptor degradation, PCSK9 inhibitors can achieve additional LDL-C reductions of approximately 50%–60% beyond the lowering achieved with statin therapy alone, providing a markedly greater lipid-lowering effect when added to background statin treatment (Gallego-Colon et al., 2020).

However, the widespread use of PCSK9 inhibitors in China remains limited. Factors such as high acquisition costs, variable reimbursement eligibility that limits coverage to selected high-risk patients, and the need for subcutaneous administration with ongoing follow-up constrain their accessibility and broader adoption in routine clinical practice. As a result, although PCSK9 inhibitors are highly effective, they are generally reserved for very-high-risk patients (e.g., those with established ASCVD or very-high-risk familial hypercholesterolemia) who remain far above LDL-C targets despite optimized oral lipid-lowering therapy (Mach, 2019).

## 3 Development history, pharmacological properties, and clinical trial evidence of hybutimibe

### 3.1 Development history of hybutimibe

The development pathway of hybutimibe reflects a full innovation-to-approval trajectory encompassing compound screening, pharmacology, toxicology, and sequential clinical evaluation. Initial compound discovery and medicinal chemistry optimization were undertaken prior to 2011. Toxicology and animal studies were conducted between 2011 and 2013, followed by a phase I clinical program (2012–2013) that characterized the drug’s safety, tolerability, and pharmacokinetic profile in humans. Phase II clinical studies were completed in 2014–2015, providing early evidence of dose–response relationships and LDL-C-lowering efficacy. A larger phase III program was conducted from 2015 to 2019 to confirm clinical benefit and safety in broader patient populations. The drug subsequently progressed to regulatory submission and received marketing approval between 2019 and 2021. This timeline underscores the systematic and rigorous development process supporting the clinical use of hybutimibe (Figure 2).

**FIGURE 2:**
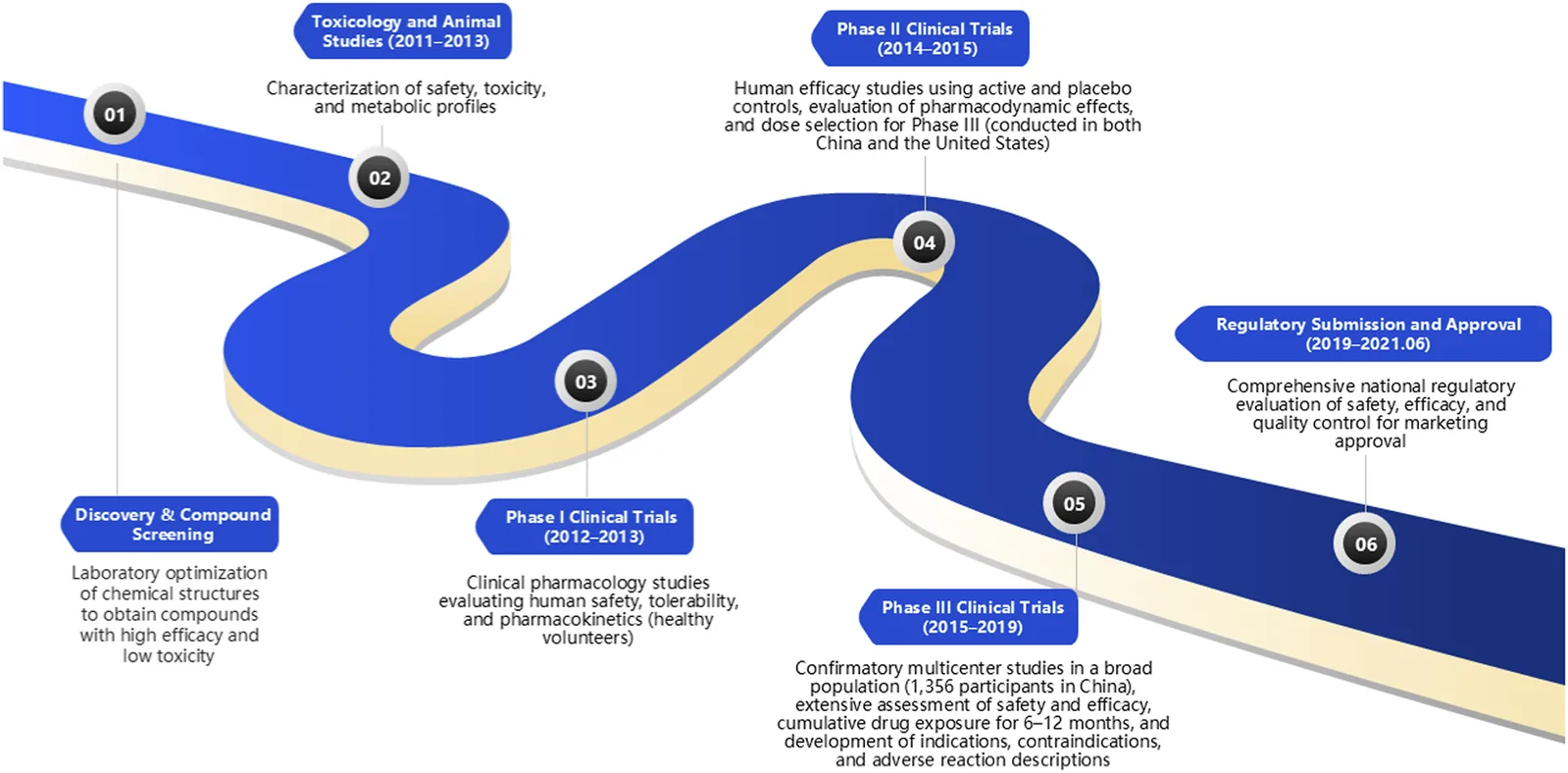
Research and Development History of Hybutimibe. The timeline shows the systematic process of hybutimibe from preclinical research to marketing application, reflecting the rigor of research and development for domestic innovative drugs. Pre-2011: Completion of compound screening and medicinal chemistry optimization, confirmation of selective NPC1L1 inhibitory activity; 2011-2013: Conduct of preclinical toxicology and animal experiments to verify safety, simultaneous initiation of Phase I clinical trials (2012–2013) to clarify pharmacokinetic characteristics and safety in healthy populations; 2014-2015: Phase II clinical trials to explore dose-effect relationship and verify preliminary lipid-lowering efficacy; 2015-2019: Phase III clinical trials to verify long-term efficacy and safety in a broad population; 2019-2021: Completion of regulatory submission and marketing approval in China, becoming the first domestic cholesterol absorption inhibitor. The timeline clearly presents the researchanddevelopment logic of hybutimibe, providing evidence-based support for its clinical application.

### 3.2 Pharmacological identity of hybutimibe

Hybutimibe inhibits NPC1L1-mediated uptake of dietary and biliary cholesterol at the intestinal brush border. Reduced delivery of intestinal cholesterol to the liver lowers the hepatic cholesterol pool and promotes upregulation of hepatic LDL receptors, thereby increasing the clearance of circulating LDL particles. However, reduced intestinal cholesterol absorption may also trigger compensatory activation of hepatic cholesterol synthesis (Altmann et al., 2004; Davis et al., 2004) (Figure 3). This compensatory response provides the biological rationale for combining an NPC1L1 inhibitor with a statin: the former reduces intestinal cholesterol entry, while the latter inhibits hepatic cholesterol synthesis through HMG-CoA reductase. The complementary effects of these mechanisms can produce greater LDL-C lowering than either pathway alone.

**FIGURE 3:**
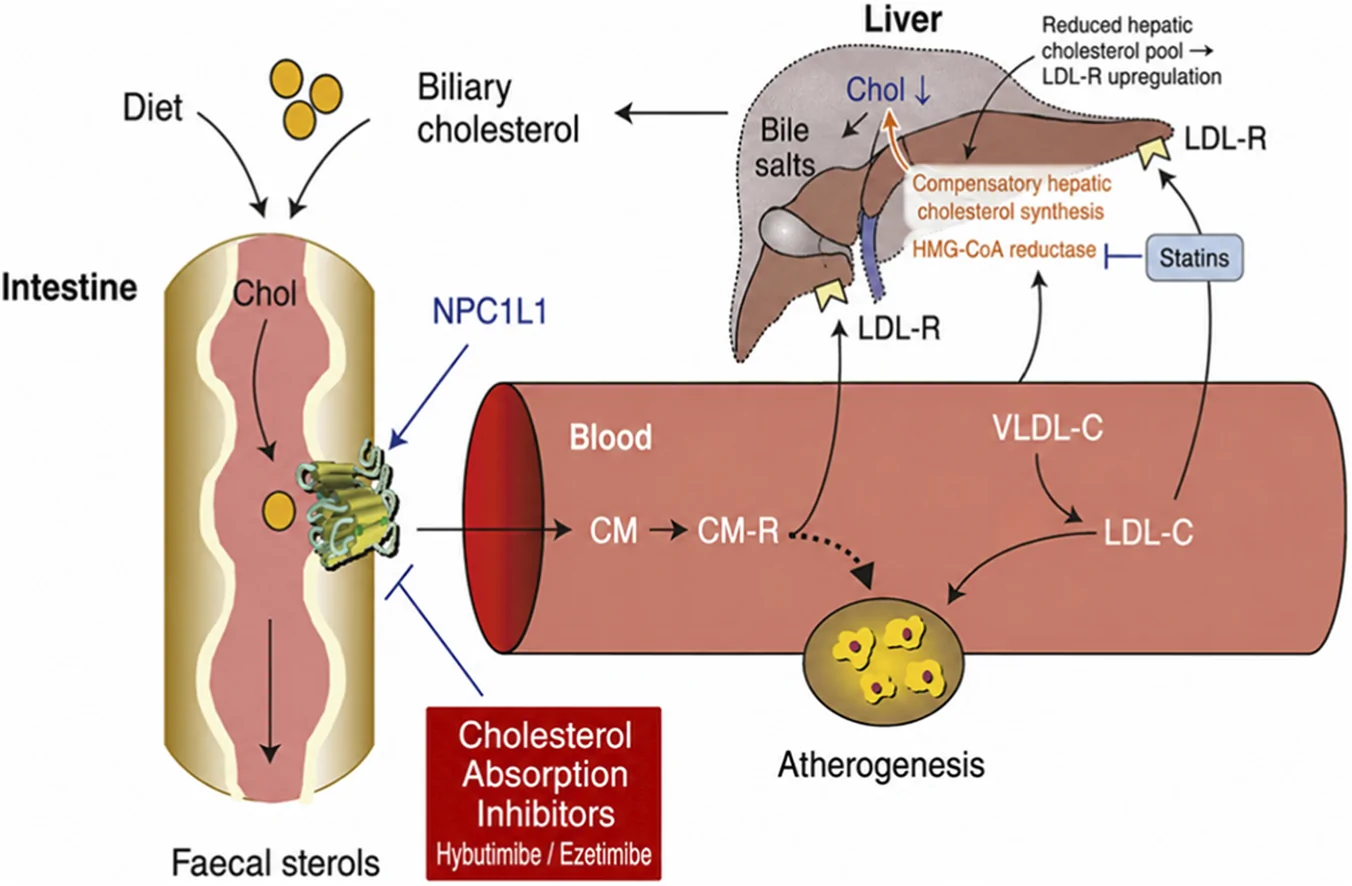
NPC1L1 Inhibition and the Mechanistic Rationale for Combination With Statins. NPC1L1 mediates the uptake of dietary and biliary cholesterol at the intestinal brush border. Inhibition of NPC1L1 reduces intestinal cholesterol delivery to the liver, decreases the hepatic cholesterol pool, and promotes LDL-receptor expression and circulating LDL clearance. Reduced intestinal cholesterol absorption may induce compensatory hepatic cholesterol synthesis through the HMG-CoA reductase pathway. Statins inhibit this compensatory pathway, providing a mechanistic rationale for combining statins with a cholesterol absorption inhibitor. These mechanistic effects support complementary LDL-C lowering but do not by themselves establish drug-specific cardiovascular outcome benefits.

### 3.3 Structural and pharmacokinetic characteristics of hybutimibe

Hybutimibe differs structurally from ezetimibe through changes in the substituent, double bond, and hydroxyl-group positions (Table 1). These modifications are associated with differences in glucuronidation, absorption, plasma protein binding, and routes of excretion. In a single-dose radiolabeled study involving six healthy male participants, hybutimibe-glucuronide accounted for 97.2% of total plasma radioactivity, and 16.39% and 76.90% of administered radioactivity were recovered in urine and feces, respectively. Because this was a small, open-label pharmacokinetic study, these findings primarily characterize drug disposition rather than comparative clinical efficacy or safety.

**TABLE 1:** Reported structural and pharmacokinetic characteristics of hybutimibe and ezetimibe.

Characteristics	Hybutimibe	Ezetimibe	Interpretation and evidence status	References
Chemical Structure	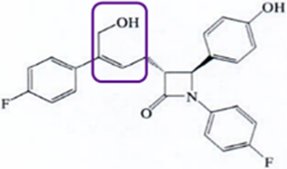	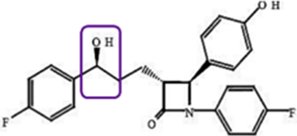	​	​
Glucuronide conjugate conversion rate	98%–99%	80%–90%	Higher metabolic conversion	​
Plasma protein binding	94.50%	88%–92%	Difference in protein binding; clinical relevance is unknown	(Ruan et al., 2014)
Time to peak concentration (Tmax)	As early as 0.5 h	∼4 h	Earlier reported Tmax; clinical onset has not been directly compared	(Liao et al., 2021)
Urinary excretion ratio	16.39%	11%	Different excretion pattern observed in separate radiolabeled studies; implications for efficacy and safety remain uncertain	​

Values were derived from separate studies and should not be interpreted as direct head-to-head comparisons. Differences in study design, dose, population, and analytical methods limit cross-study inference.

Reported pharmacokinetic parameters for hybutimibe and ezetimibe were obtained from separate studies with different designs, analytical methods, doses, and populations. Consequently, differences in time to peak concentration, protein binding, glucuronidation, or excretion should not be interpreted as evidence that hybutimibe has a faster clinical onset, more stable therapeutic exposure, less clinically relevant accumulation, or superior long-term safety. Direct head-to-head studies are required to determine whether these pharmacokinetic differences translate into clinically meaningful benefits. The available pharmacokinetic comparisons are hypothesis-generating and should not be used to support therapeutic superiority.

### 3.4 Clinical development and key findings from phase I-III trials of hybutimibe

The clinical evaluation of hybutimibe has progressed through a structured series of Phase I, II, and III studies that collectively characterize its safety, pharmacokinetic properties, and LDL-C–lowering efficacy (Table 2). Evidence from Phase I–III trials should be interpreted according to study phase, sample size, comparator, duration, and endpoint selection. Early-phase safety studies in healthy volunteers cannot establish long-term safety in patients with ASCVD, while LDL-C reduction in Phase II and III trials does not directly demonstrate cardiovascular event reduction. The first-in-human study conducted by Ruan et al. (Ruan et al., 2014) assessed single ascending doses (1, 3, 5, 10, 20, and 30 mg) and multiple ascending doses (5, 10, and 20 mg) in healthy volunteers. This study demonstrated favorable safety and tolerability across all dose levels. It also established the fundamental pharmacokinetic profile of hybutimibe, including the influence of food intake, and showed that the agent produced measurable lipid-lowering effects even in healthy subjects, supporting its potential as a therapeutic candidate for hypercholesterolemia.

**TABLE 2:** Phase I-III clinical studies of hybutimibe.

Study type	Design and sample size	Population	Intervention	Follow-up duration	Key efficacy findings	Safety profile
Single-center first-in-human (dose-escalation study) (Altmann et al., 2004)	First-in-human, single-center, single- and multiple-ascending-dose and food-effect study; 5–10 hybutimibe-treated participants per single-dose cohort	Healthy volunteers	Single ascending doses (1, 3, 5, 10, 20, 30 mg) and multiple ascending doses (5, 10, 20 mg) of hybutimibe	Single dosing and 10-day repeated dosing	–	All adverse events were mild to moderate; no clinically significant laboratory abnormalities; most subjects had no adverse events
Phase I randomized, double-blind, placebo-controlled dose-escalation study	Randomized, double-blind, placebo-controlled, parallel-group Phase I study	Healthy adults in China and the United States	Single oral doses of 1–30 mg; repeated dosing with 5, 10, or 20 mg once daily for 10 days	10 days (treatment period)	–	Single and multiple doses were well tolerated; most adverse events were mild; overall safety favorable
Phase II multicenter, randomized, double-blind, placebo- and active-controlled study (Liao et al., 2021)	Multicenter, randomized, double-blind, placebo- and positive-controlled, parallel-group Phase II study	Patients with primary hyperlipidemia	Hybutimibe ≥5 mg once daily	8 weeks	Significant reductions in LDL-C, TC, non-HDL-C, and ApoB; no significant effect on TG	Overall well tolerated; most adverse events were mild; elevated liver enzymes and gastrointestinal reactions were infrequent; severe adverse events were rare (1.03%); no drug-related serious adverse events
Phase III multicenter monotherapy study (Ruan et al., 2014)	Multicenter, randomized, double-blind, placebo-controlled, parallel-group Phase III study; n = 373	Patients with primary hypercholesterolemia	Hybutimibe monotherapy	12-week placebo-controlled period plus 40-week extension; 52 weeks in total	Significant LDL-C reduction; lipid-lowering effect sustained; treatment well tolerated	Low incidence of adverse events; most were mild; withdrawal rate <5%; no new safety signals
Phase III multicenter randomized, double-blind combination-therapy study (Qi et al., 2022a)	Multicenter, randomized, double-blind, placebo-controlled, parallel-group Phase III study; n = 255	Patients with hypercholesterolemia and ASCVD	Hybutimibe 20 mg plus atorvastatin 10 mg versus atorvastatin 20 mg	12 weeks	Mean LDL-C changes from baseline at week 12 were −16.39% and −6.75%, respectively (P < 0.0001)	Adverse-event incidence low; no serious drug-related events; discontinuation rate low; safety profile consistent with Phase III data
Phase III multicenter monotherapy or combination therapy study (Schwartz et al., 2018)	Multicenter, randomized, double-blind, double-dummy, parallel-controlled Phase III study; n = 727	Patients with primary hypercholesterolemia	Hybutimibe monotherapy or combination with atorvastatin	12-week treatment period plus 40-week long-term evaluation; 52 weeks in total	Combination therapy further improved LDL-C reduction compared with monotherapy	No new drug-related severe adverse events within 52 weeks; overall tolerability excellent

Additional Phase I studies performed in healthy adults in both China and the United States further confirmed these findings. In a randomized, double-blind, placebo-controlled, parallel-group study, single oral doses ranging from 1 to 30 mg were well tolerated in all participants. Repeated once-daily dosing of 5, 10, or 20 mg for 10 days also demonstrated consistent safety and good tolerability across populations, reinforcing the favorable early-phase safety profile of hybutimibe.

The efficacy of hybutimibe was subsequently explored in a multicenter, randomized, double-blind, placebo- and active-controlled Phase II study involving patients with primary hyperlipidemia (Qi et al., 2022b). Treatment with hybutimibe at a minimum daily dose of 5 mg significantly reduced LDL-C, total cholesterol (TC), non high density lipoprotein-cholesterol (non HDL-C), and apolipoprotein B (apoB). The drug was well tolerated throughout the study period, and no significant effect on triglyceride levels was observed.

Phase III investigations provided more extensive evidence for the lipid-lowering efficacy and long-term safety of hybutimibe. In a multicenter monotherapy study, hybutimibe significantly reduced LDL-C in patients with primary hypercholesterolemia and demonstrated sustained lipid-lowering effects over long-term therapy with a low incidence of adverse events and good overall tolerability (Chen, 2022). A separate multicenter, randomized, double-blind Phase III study evaluated hybutimibe in patients with hypercholesterolemia and comorbid ASCVD or other high-risk conditions. Among individuals whose LDL-C levels remained above target despite atorvastatin 10 mg daily, the addition of hybutimibe 20 mg produced an additional 16.39% reduction in LDL-C, which was substantially greater than the 6.75% reduction achieved by doubling the atorvastatin dose (Qi et al., 2022a) (Figure 4).

**FIGURE 4:**
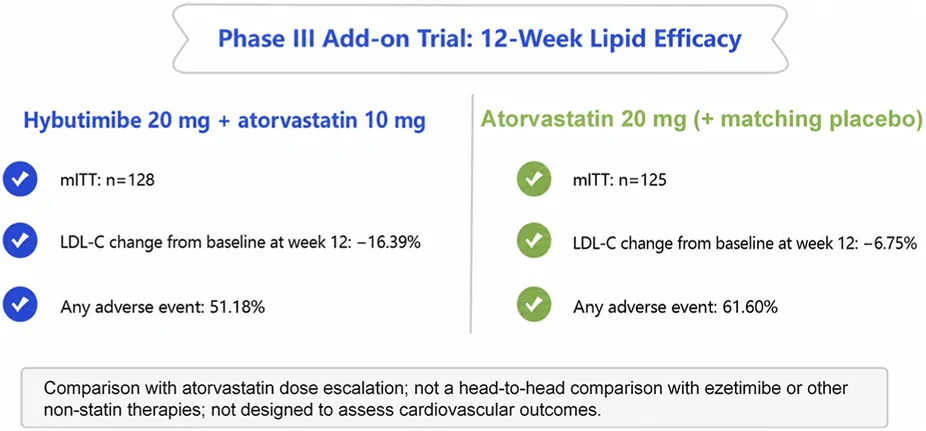
LDL-C-Lowering Strategies Evaluated in the Phase III Add-on Trial of Hybutimibe. In patients whose LDL-C remained above target during atorvastatin 10 mg/day therapy, add-on hybutimibe 20 mg/day was compared with atorvastatin dose escalation over 12 weeks. The additional LDL-C reductions were 16.39% and 6.75%, respectively. The trial supports greater short-term LDL-C lowering with add-on hybutimibe than with atorvastatin dose escalation under the studied conditions. It did not compare hybutimibe with ezetimibe or other contemporary non-statin therapies and was not designed to evaluate cardiovascular outcomes.

Further Phase III evidence demonstrated that hybutimibe used alone or in combination with atorvastatin provides reliable LDL-C reduction and maintains a favorable safety profile in patients with primary hypercholesterolemia (Cai, 2023). This body of work supports the role of hybutimibe as an effective adjunct to statins, particularly in patients who do not achieve LDL-C goals with statin monotherapy.

The clinical development program therefore supports the LDL-C-lowering efficacy of hybutimibe over treatment periods of up to 52 weeks. However, the available trials were not designed or powered to evaluate major adverse cardiovascular events, cardiovascular mortality, or all-cause mortality. The cardiovascular benefit established for LDL-C lowering as a therapeutic principle cannot substitute for drug-specific cardiovascular outcome evidence. Whether the lipid effects of hybutimibe translate into improved clinical outcomes remains to be determined.

### 3.5 Safety, tolerability, and drug-interaction evidence

Across Phase I–III studies, reported adverse events were generally mild to moderate (Qi et al., 2022a; Cai, 2023; Chen, 2022), but the duration and sample size of these studies limit assessment of uncommon or delayed adverse reactions. In the Phase II study, the most frequently reported events included elevations in alanine aminotransferase, gastrointestinal reactions, dizziness, and headache (Qi et al., 2022a). Available Phase III data did not identify treatment-related deaths or new short-term safety signals, although overall adverse-event rates were not consistently lower with hybutimibe-containing regimens (Qi et al., 2022a; Cai, 2023; Chen, 2022).

Evidence regarding muscle-related symptoms, clinically significant creatine kinase elevation, persistent hepatic enzyme elevation, treatment discontinuation, renal impairment, and drug–drug interactions remains limited. In particular, the absence of a reported interaction in existing trials should not be interpreted as evidence that clinically relevant interactions have been excluded. Larger pharmacovigilance datasets and dedicated interaction studies are needed to characterize long-term safety in patients receiving multiple cardiovascular and metabolic medications.

## 4 Real-world efficacy, hepatic impairment safety profile, and clinical value of hybutimibe

### 4.1 Use in patients with hepatic impairment: pharmacokinetic and safety considerations

Post-marketing evaluations have provided additional insight into the clinical utility of hybutimibe, particularly in patient groups for whom lipid-lowering therapy is often constrained by safety considerations. In China, metabolic dysfunction–associated fatty liver disease is common and a meaningful proportion of patients present with mild or moderate hepatic impairment. Statin therapy in such individuals may be limited by elevations in liver enzymes, which restrict the ability to intensify LDL-C-lowering regimens. Pharmacokinetic studies have shown that hybutimibe does not require dose adjustment in patients with mild or moderate hepatic dysfunction. The observed increases in total drug exposure, approximately 1.3-fold and 1.5-fold respectively, remain within the two-fold threshold recommended by the CDE guidance. By comparison, ezetimibe exposure can increase as much as four-fold in moderate hepatic impairment, and its prescribing information indicates that it is not recommended in this population. These pharmacokinetic observations suggest that hybutimibe may warrant further evaluation in patients with mild or moderate hepatic impairment. However, the available evidence does not establish superior clinical safety over ezetimibe, and treatment decisions should remain consistent with the approved prescribing information and individual hepatic status.

### 4.2 Real-world effectiveness and safety across high-risk populations

Several observational studies have reported lipid and clinical parameters among Chinese patients receiving hybutimibe-containing regimens. These studies provide preliminary information on use in routine practice but should not be assigned the same evidentiary weight as randomized controlled trials because of their retrospective designs, limited sample sizes, short follow-up, heterogeneous background therapy, and potential residual confounding (Liu et al., 2024).

In patients with coronary heart disease, a retrospective study showed that adding hybutimibe to atorvastatin plus evolocumab was associated with a higher investigator-defined response rate (80.00% vs. 62.50%) after 4 weeks and resulted in lower post-treatment lipid levels, including TC (3.01 ± 0.47 mmol/L), TG (1.01 ± 0.26 mmol/L), LDL-C (1.08 ± 0.24 mmol/L) and Lp(a) (187.16 ± 8.12 mg/L), without evidence of worsened hepatic or renal function or increased adverse events (Wang and Xu, 2025).

In elderly patients with chronic heart failure and hyperlipidemia, combination therapy with sacubitril/valsartan and hybutimibe was associated with changes in lipid parameters and measures of cardiac function, and has been associated with a lower 6-month event rate, although the study was not designed to establish causality (P < 0.05) (Yunxiang et al., 2025). In another coronary heart disease cohort, hybutimibe added to atorvastatin achieved greater reductions in LDL-C, TC and TG than atorvastatin alone, and was also associated with improvements in vascular structure, including lower carotid intima–media thickness and plaque area; adverse event rates were comparable between groups (7.89% vs. 11.43%) (Mu and Qiu, 2025). Among patients with large-artery atherosclerotic ischemic stroke, hybutimibe combined with atorvastatin was associated with further reductions in LDL-C and imaging measures of plaque burden, together with a lower 1-year recurrence rate than statin monotherapy; these findings require confirmation in a randomized outcome trial (Wu et al., 2025).

The generalizability of these findings is limited. Most post-marketing studies were conducted in China, and many were retrospective, single-center, relatively small, and published in regional journals. Differences in ethnicity, dietary patterns, background lipid-lowering therapy, comorbidities, reimbursement systems, and clinical practice may influence treatment response and implementation. Validation in prospectively designed multinational and multiethnic cohorts is required before these findings can be generalized to broader international populations.

### 4.3 Potential clinical positioning

Current evidence supports hybutimibe as an oral LDL-C-lowering option in China, either as monotherapy or in combination with a statin. A potential use is in patients who remain above LDL-C targets despite a tolerated statin regimen or in whom further statin dose escalation is undesirable. However, the available evidence does not establish that hybutimibe should be preferred over or routinely replace ezetimibe.

Clinical selection among non-statin therapies should consider the required magnitude of LDL-C reduction, cardiovascular outcome evidence, route and frequency of administration, tolerability, cost, reimbursement, and patient preference. PCSK9 monoclonal antibodies provide greater LDL-C reduction and have cardiovascular outcome evidence but require injection and may be limited by cost and access (Sabatine et al., 2017; Schwartz et al., 2018). Bempedoic acid provides an oral option with cardiovascular outcome evidence in statin-intolerant patients (Nissen et al., 2023), whereas inclisiran offers infrequent administration but awaits definitive cardiovascular outcome evidence (Ray et al., 2020). Formal cost-effectiveness analyses and direct comparative trials are needed before the relative position of hybutimibe can be defined in international treatment algorithms.

## 5 Knowledge gaps and limitations of the current evidence

Several limitations define the current evidence base for hybutimibe. First, no dedicated cardiovascular outcome trial has determined whether its LDL-C-lowering effect reduces major adverse cardiovascular events, cardiovascular mortality, or all-cause mortality. Second, the pivotal trials primarily used lipid parameters as surrogate endpoints, included relatively limited numbers of participants, and had follow-up periods of no more than 52 weeks. Third, direct comparative evidence against ezetimibe, bempedoic acid, inclisiran, or PCSK9 monoclonal antibodies is insufficient to establish relative efficacy, safety, or cost-effectiveness.

In addition, most randomized and observational studies were conducted in Chinese populations. The real-world evidence is particularly limited by retrospective designs, small or single-center cohorts, short follow-up, and potential residual confounding. Evidence regarding long-term hepatic and muscle safety, renal impairment, drug–drug interactions, treatment persistence, and multiethnic populations remains incomplete. These limitations should be considered when interpreting the current clinical role and international applicability of hybutimibe.

## 6 Future research directions

Future research should prioritize adequately powered cardiovascular outcome trials evaluating major adverse cardiovascular events, cardiovascular mortality, and all-cause mortality. Longer post-marketing follow-up is also needed to identify uncommon or delayed adverse events and to characterize safety in older adults, patients with hepatic or renal impairment, and patients receiving multiple cardiovascular and metabolic therapies.

Additional priorities include multinational randomized trials, direct comparisons with ezetimibe and other contemporary non-statin therapies, pharmacoeconomic and budget-impact analyses, and evaluation of treatment response across different ethnic and geographic populations. Studies of fixed-dose combinations should assess not only pharmacokinetics and LDL-C lowering but also adherence, persistence, safety, and clinically relevant outcomes. Biomarkers of cholesterol absorption and synthesis may also help determine whether specific metabolic phenotypes predict response to NPC1L1 inhibition.

## 7 Conclusion

Hybutimibe is an oral NPC1L1 inhibitor with demonstrated LDL-C-lowering efficacy as monotherapy and in combination with statins in predominantly Chinese clinical trial populations. Available studies suggest generally acceptable short-to medium-term tolerability, but they have primarily evaluated lipid surrogate endpoints and have follow-up periods of no more than 52 weeks. The reported pharmacokinetic differences between hybutimibe and ezetimibe have not been shown to confer superior clinical efficacy, safety, or cardiovascular outcomes.

The absence of dedicated cardiovascular outcome trials, limited multinational evidence, insufficient direct comparison with contemporary non-statin therapies, and relatively limited long-term post-marketing data currently constrain its broader clinical positioning. Larger multinational randomized trials, long-term pharmacovigilance, comparative-effectiveness studies, and cardiovascular outcome trials are required to define its role in contemporary lipid management.
